# Microstructure and Thermal Stability of Cu/Ti_x_Si_y_N/AlSiN Solar Selective Absorbing Coating

**DOI:** 10.3390/ma13040882

**Published:** 2020-02-16

**Authors:** Hongwen Yu, Yanli Zhang, Qian Zhang, Wei Pang, Hui Yan, Guangyuan Li

**Affiliations:** 1 College of Materials Science and Engineering, Key Laboratory of Advanced Functional Materials of Education Ministry of China, Beijing Key Laboratory of Microstructure and Property of Advanced Materials, Beijing University of Technology, Beijing 100124, China; hongwen_yu@163.com (H.Y.); 13646399507@163.com (Q.Z.); pw724976911@163.com (W.P.); 2 School of Statistics, Shandong University of Finance and Economics, Jinan 250014, China; zhangylhappy@163.com; 3 Energy Research Institute, Qilu University of Technology (Shandong Academy of Sciences), Jinan 250014, China

**Keywords:** solar selective coatings (SSC), magnetron sputtering, (Ti,Si)N, thermal stability

## Abstract

An oxygen-free solar selective absorbing coating of Cu/Ti_x_Si_y_N/AlSiN was prepared on a Cu buffered stainless steel substrate by magnetron sputtering. This coating was prepared using a single target for each layer. A spectrophotometer, Fourier transform infrared spectrophotometer, scanning electron microscopy, X-ray diffractometer and atomic force microscopy were used to characterize the optical properties, crystalline structure, morphology and composition of these coatings. The coating of Cu/Ti_x_Si_y_N/AlSiN has good optical properties (average absorption of 0.941 and emittance of 0.058) and excellent thermal stability. The performance criterion (PC) is 0.0365, when the solar selective absorbing coating is heated in air at 200 °C for 1200 h.

## 1. Introduction

As the world faces severe energy challenges [1], it is necessary to respond urgently by cutting greenhouse gas emissions in order to achieve the goals of the Paris Agreement on climate change [2,3]. The development and implementation of non-fossil energy technologies has become increasingly important [4]. Solar energy is both a primary energy source and a clean renewable energy source. With its unique advantages, it has become a very ideal energy source to replace conventional fossil fuels [2,4].

Based on a 2017 global energy consumption analysis, industrial energy accounts for 32% of global energy consumption, of which 74% is industrial heat, but currently only 9% of industrial heat is provided by renewable energy [5]. Thirty percent of industrial heat is below 150 °C [5], which can be provided or supplemented by solar energy; therefore solar-thermal utilization has great potential in the market.

Solar–thermal utilization uses a special coating to directly convert solar energy into heat [6], and the efficiency is generally above 60% [7]. Researchers have used solar concentrators, such as Fresnel collectors [8], parabolic trough collectors [9], etc., or changed the structure of collectors, such as double-cover collectors [10], to achieve mid-temperature utilization of solar energy (80–250 °C). These structurally changed collectors solve the problems of solar radiation gap and low density, in order to achieve the medium temperature heat utilization of solar energy. In addition to the previous structural improvements, it is important to study the long-term thermal stability of solar selective coatings (SSC) [11,12].

**Figure 1:**
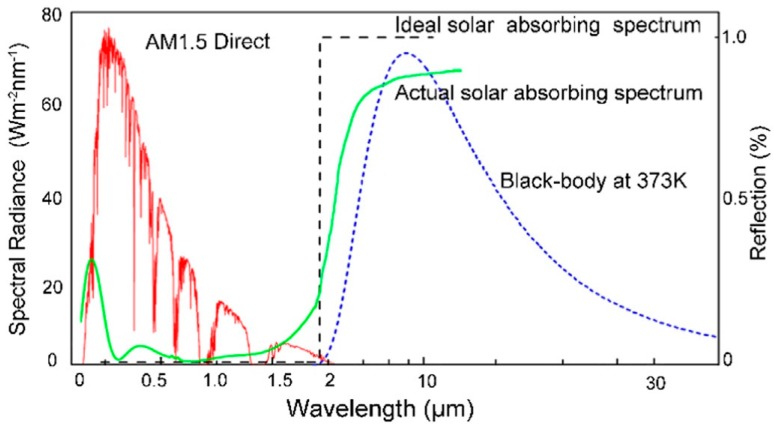
The ideal solar selective absorption spectrum.

Solar radiation has a wide spectral range, with energy mainly concentrated in the range of ultraviolet, visible and near infrared (300–3000 nm) [12]. Generally, the thermal radiation wavelength of the absorber is greater than 3 μm. The ideal selective absorption coating should have the maximum solar absorptivity in the energy concentrated spectral range (300–2500 nm) and minimal thermal emissivity [1]. This characteristic can be expressed by solar selectivity (S=α/ε) [13]. Figure 1 clearly shows the ideal solar selective absorption spectrum, black body radiation spectrum, actual solar selective absorption spectrum and solar radiation spectrum (AM1.5). Moreover, SSC should be thermally stable given its severe working conditions [14]. A wide variety of SSC have been developed in the past to meet these requirements.

The preparation of high-temperature coating is mostly made by magnetron sputtering, mainly using metal particles as the absorption units in metal–dielectric cermet coatings, such as Cu/TiN_x_O_y_/TiO_2_/Si_3_N_4_/SiO_2_ [15], Pt/Al_x_O_y_ [16], Al_y_Ti_1−y_(O_x_N_1−x_) [17], AlCrSiN/AlCrSiON/AlCrO [18], etc., which have excellent optical characteristics and thermal stability [19]. However, the use of oxygen to prepare oxide or nitrogen oxide coatings can cause the oil in the vacuum pump to oxidize [17,20], reducing its pumping speed and service life, and even affecting product quality and production efficiency [21]. To improve this issue, under current equipment conditions, we use nitrides of transition metals as an oxygen-free coating.

In this work, we apply magnetron sputtering to prepare an oxygen-free coating of Cu/Ti_x_Si_y_N/AlSiN on a stainless steel (SS) substrate. The entire coating stack was completed with a pure metallic copper (Cu) layer at the substrate coating as the infrared reflection layer and the binding layer and interface to provide high reflectivity (and low emissivity) in the infrared region. Ti_x_Si_y_N is known for its high thermal stability [22] and exceptional corrosion resistance. Ti_x_Si_y_N is used as an absorbing layer. Absorbing layers with different metal volumes are prepared by adjusting the nitrogen content and changing optical parameters. AlSiN acts as an anti-reflection layer and reduces the diffusion of oxygen and other pollutants from the surrounding atmosphere into the coating [23]. The biggest feature of our oxygen-free coating preparation process is that only one target is required for each layer, which has better manufacturability and is suitable for continuous large-scale production.

## 2. Experiment Details

All targets with a diameter of 75 mm were commercially purchased, Cu target (LOYAL, Beijing, China) purity 99.99%, Ti/Si (Ti90Si10 wt%) and Al/Si (Al90Si10 wt%) (Okai, Baoji, China) purity 99.995% and grain size 30–50 nm. Prior to being loaded into the vacuum chamber, a stainless steel (SUS304-2B) substrate (Ø30 mm) was cleaned in an ultrasonic bath using ethanol for 15 min. Then the deposition started at a base pressure of 7 × 10^−4^ Pa; the substrate was cleaned by ionic sputtering using an Ar^+^ plasma to remove grease, the native oxide layer and the physisorbed layers. The multilayer coatings were synthesized by depositing each layer successively. There was an exception regarding the Cu layer, which was predeposited on the SS substrate at 250 °C for 15 min. Table 1 lists the optimal process parameters, including sputtering pressure, gas flow rate, sputtering time, etc. The criteria for optimization included solar absorptance (α), thermal emittance (ε) and solar selectivity (S) of the as-deposited SSC.

A scanning electron microscope (Hitachi, S5200, Tokyo, Japan) equipped with a field emission gun (FEG) was employed to analyze the morphology of the deposited samples (SEM). Cross-section images of the samples deposited on SS substrates were measured at 1 and 5 kV electron beam energy. The phase structure of the thin films was determined by X-ray diffraction employing grazing incidence geometry (GIXRD) using a Rigaku Ultima IV diffractometer (D8 ADVANCE, Bruker, Ettlingen, Germany) with Cu-Kα radiation (λ = 1.5406 Å). The incident angle was 0.4°, and the XRD patterns were measured in the diffraction angle range of 20°–100° in steps of 0.02°. Interfacial bonding strength was quantitatively measured by the scratch test (WS-2004, automatic coating adhesion tester, Nanjing, China). Microstructures of the SSC were analyzed by using atomic force microscopy (Zhuolun Micronano, MicroNano D3000, Shanghai, China). A UV-Vis-NIR (Shimadzu, UV-3600, Kyoto, Japan) spectrophotometer was used to measure the reflectance spectra of the SSC at room temperature with an AM 1.5 reference spectrum. The wavelength range was set between 300 and 2500 nm and was corrected using a BaSO_4_ baseline sample, which was provided by the manufacturer of the spectrophotometer. The thermal emittance (*ε*) measurements were carried out by a Fourier transform infrared (FTIR) spectrometer (Shimadzu, IR-Affinity-1, Kyoto, Japan). The measurement range was 400 to 4000 cm^−1^, and the resolution was 0.4 cm^−1^. Solar absorptance (α) and thermal emittance (ε) were calculated using Equations (1) and (2) (specification for absorber of flat plate solar collector, GB/T 26974-2011, China), respectively:

(1) $$\alpha =\frac{\int_{300\mathrm{nm}}^{2500\mathrm{nm}}\left[1-R(\lambda )\right]P_{\mathrm{sun}}(\lambda )d\lambda }{\int_{300\mathrm{nm}}^{2500\mathrm{nm}}P_{\mathrm{sun}}(\lambda )d\lambda }$$

(2) $$\varepsilon =\frac{\int_{300\mathrm{nm}}^{2500\mathrm{nm}}\left[1-R(\lambda )\right]P_{B}(\lambda )d\lambda }{\int_{300\mathrm{nm}}^{2500\mathrm{nm}}P_{B}(\lambda )d\lambda }$$

where R(λ) is the reflectance, $P_{\mathrm{sun}}\left(\lambda \right)$ is the intensity of incident AM 1.5 solar radiation and $P_{B}(\lambda )d\lambda$ is Planck blackbody radiation spectrum.

We carried out ageing tests and evaluated the performance changes in order to elucidate the durability at high temperatures in air [24]. We evaluated the performance criterion (PC) parameter after each annealing cycle. According to the GB/T 26974-2011 measurement conditions, the sample was heated in air at 200 °C for no less than 200 h. The PC of the coating can be expressed by the following relation:

(3) PC=−Δα+0.5Δε

where Δα is the change in the solar absorptivity defined as the difference between the value at the actual time of the test and the initial value of solar absorptivity, and Δε is the change in the thermal emissivity.

The SSC was subjected to annealing tests in air at 200 °C for 200, 400, 800 and 1200 h. To test thermal stability after each step, the coating was allowed to return to a normal temperature, and the reflectance was measured to evaluate the absorptance and the emittance.

## 3. Results and Discussions

### 3.1. Microstructure and Optical Characterization

**Figure 2:**
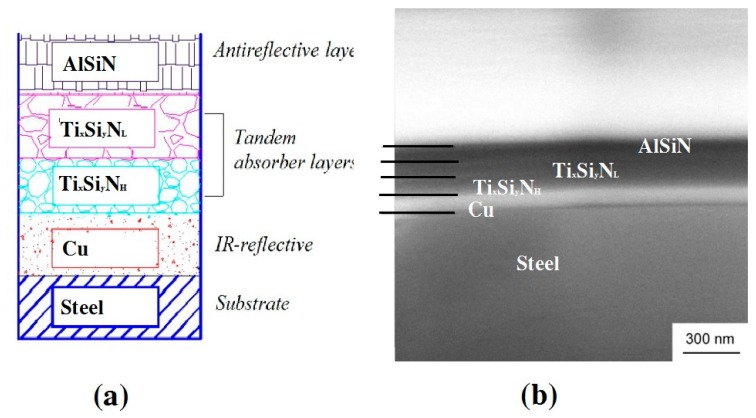
(**a**) Schematic diagram, and (**b**) cross-section microstructure of solar selective coatings (SSC).

We adopted a coating structure design using a typical double cermet coating, as shown in Figure 2a. The difference was that the high metal volume fraction (HMVF) and low metal volume fraction (LMVF) cermet absorption layers were realized by the same target, and different optical parameters were reflected by controlling different nitrogen contents. This coating was, not only effectively absorbed by the cermet matrix [25], but also by phase interference in the double cermet coating. Figure 2b shows the cross-section structure of a stack deposited on the SS substrate according to the optimization. The aim was to arrive at the calculated optimal thicknesses by using the deposition rates calibrated from the previous depositions of individual layers. It should be noted that the contrast in SEM between the three nitride layers was poor due to the small difference in composition. However, careful image analysis enabled the identification of layer boundaries such that the thickness of the layers could be determined within ±5 nm. The thicknesses of Cu, Ti_x_Si_y_N_H_, TixSiyN_L_ and AlSiN were found to be 100, 80, 70 and 70 nm, respectively.

**Figure 3:**
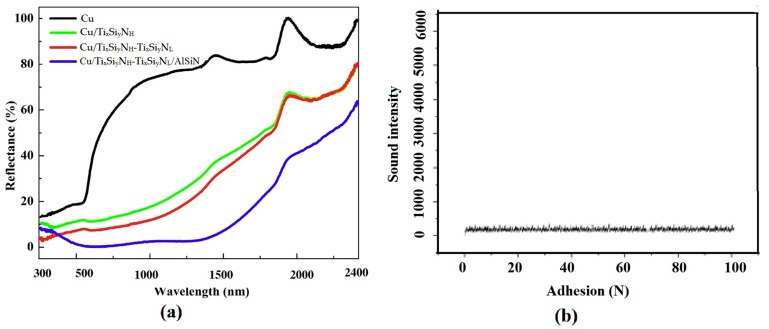
(**a**) Reflectance of different layers and (**b**) coating adhesion strength test.

The reflection spectra of Cu, Cu/Ti_x_Si_y_N_H_, Cu/Ti_x_Si_y_N_H_-Ti_x_Si_y_N_L_ and Cu/Ti_x_Si_y_N_H_-Ti_x_Si_y_N_L_/AlSiN prepared on the SS substrate are shown in Figure 3a. Measurements of the sample’s absorption, emittance, and solar selectivity are listed in Table 2. After layer-by-layer deposition, the absorption gradually increased. After the deposition of Ti_x_Si_y_N_H_ (HMVF) and Ti_x_Si_y_N_L_ (LMVF) in particular, the absorption increased significantly, reflecting the characteristics of good interference coatings. This shows that Ti_x_Si_y_N could achieve different spectral optical constants through different metal content volumes. Covered with an AlSiN anti-reflection layer, the absorption of the series absorber reached 0.934, indicating that AlSiN had a good anti-reflection and reflection reduction effect. At the same time, Ti_x_Si_y_N/AlSiN (“control”) coating was prepared on the SS substrate with the same parameters and compared. The absorption of the control sample was 0.958, which was slightly higher than that of the Cu-buffered SSC, but the emittance of the control sample was very high (~0.150). The results show that the Cu buffer layer in the preparation of the SS substrate by PVD to prepare SSC, played an important role in reducing the thermal radiation of the coating [18].

Interfacial bonding strength is one of the most important properties of the coating [26]. The wear resistance, oxidation resistance and service life of the coating are directly related to it. A scratch test is a quantitative method for testing interfacial bonding strength [26]. The critical load appears on the acoustic signal curve as the start of the spike. Figure 3b shows that during the increase of N from 0 to 100 N (N_max_ = 100 N, maximum load of automatic scratch tester for coating adhesion of WS-2004), the acoustic signal curve is basically a smooth curve, and no sudden peak appears. It shows that the coating had excellent adhesion on stainless steel substrate.

The reproducibility of eight samples deposited under the same conditions and the repeatability of solar-thermal properties of Cu/Ti_x_Si_y_N/AlSiN was investigated. Table 3 lists the spectral properties of these samples. The absorptance of the eight samples was narrowly dispersed between 0.932 and 0.948, while their emittance varied between 0.044 and 0.075. It is speculated that this may be caused by some errors in the nitrogen flow control during the preparation of different batches. Nevertheless, all these solar selectivity values are much higher than the Chinese standard set at not less than 0.92 (GB/T 26974-2011). Therefore, this process of preparing Cu/Ti_x_Si_y_N/AlSiN with a single target per layer has good repeatability and commercial promotion value.

**Figure 4:**
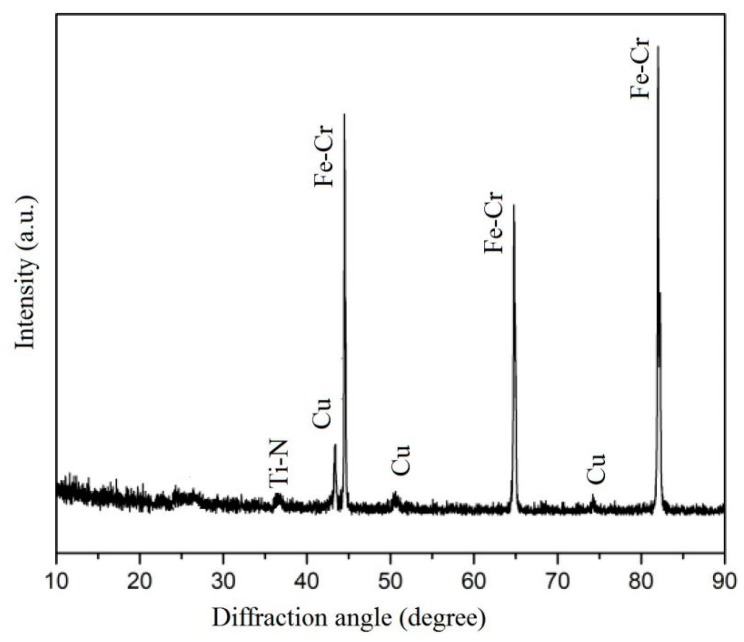
XRD 2θ scan pattern of an as-grown Cu/Ti_x_Si_y_N/AlSiN on a stainless steel (SS) substrate.

The results of the XRD patterns of SSC deposited on polished stainless steel are shown in Figure 4. The as-deposited coating contained a small amount of crystalline phase, which showed a cubic TiN-like structure with a preferred orientation of (111). The peaks of Fe-Cr correspond to the stainless steel substrate. The Cu layer prepared showed a polycrystalline structure. On the other hand, TixSiyN and AlSiN showed no characteristic XRD peaks except those of the SS substrate, suggesting a dominating amorphous or poorly crystallized microstructure for these layers. 

### 3.2. Thermal Stability

**Figure 5:**
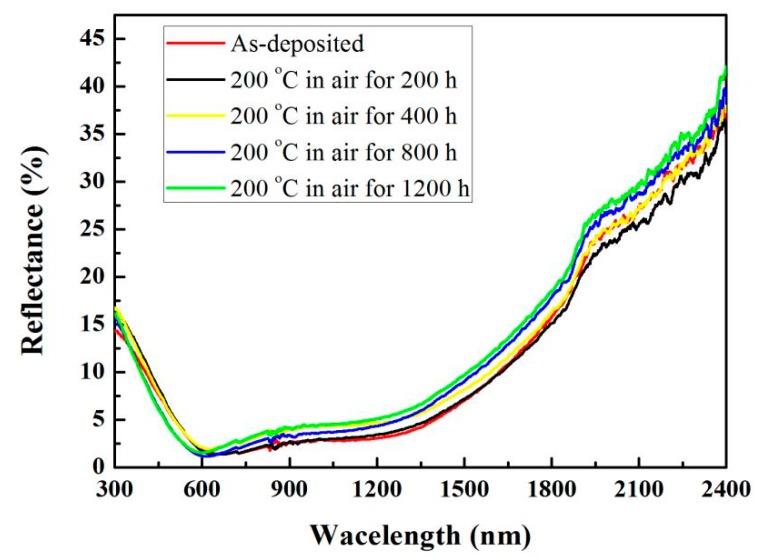
Reflectance of coating after heat treatment.

The thermal stability of the coating was evaluated by heat treatment in air [10]. Table 4 lists the solar absorption, emittance and PC after heat treatment. When the coating is heat treated for 200 h (GB/T 26974-2011), the PC of SSC is 0.0065 < 0.05. The long-term test for 1200 h, the PC is 0.0365, indicating that the coating has thermal stability beyond the standard. Figure 5 shows the reflectance of the coating after heat treatment. After the heat treatment, the absorption of the coating remains almost unchanged, and the PC change is mainly caused by the increase of the emittance. After the heat treatment, the reflectance slowly increases in the infrared band with the heat treatment time.

**Figure 6:**
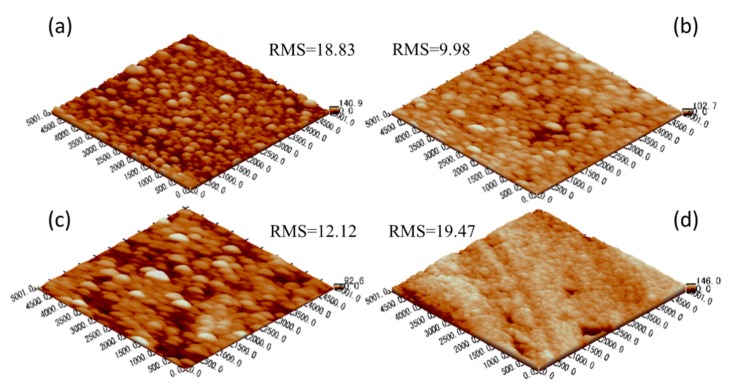
3D AFM images of the coatings, (**a**) as-deposited after heat treatment at 200 °C for (**b**) 200 h, (**c**) 800 h and (**d**) 1200 h.

Figure 6 shows atomic force microscopy (AFM) images of the coating after heat treatment. Compared with the as-deposited image, the average grain size of the coating surface changes little, so the Root Mean Square (RMS) surface roughness changes little, indicating that the coating structure is stable. This observation is consistent with the thermal stability results shown in Table 4 and Figure 4. According to these results, it can be found that the Ti_x_Si_y_N_H_ (HMVF) and AlSiN (anti-reflection) layers play a good protective coating role, and no obvious pollutants diffuse into the absorbing layer, which seriously affects the optical characteristics.

In the course of the experiment, we occasionally found oxygen in the coatings, but the probability of occurrence was irregular, and the percentage of oxygen was not fixed. In comparing coatings with a small amount and those which were oxygen-free, we found that trace oxygen in the coating had no significant effect on the reflectance spectrum of the coating.

**Figure 7:**
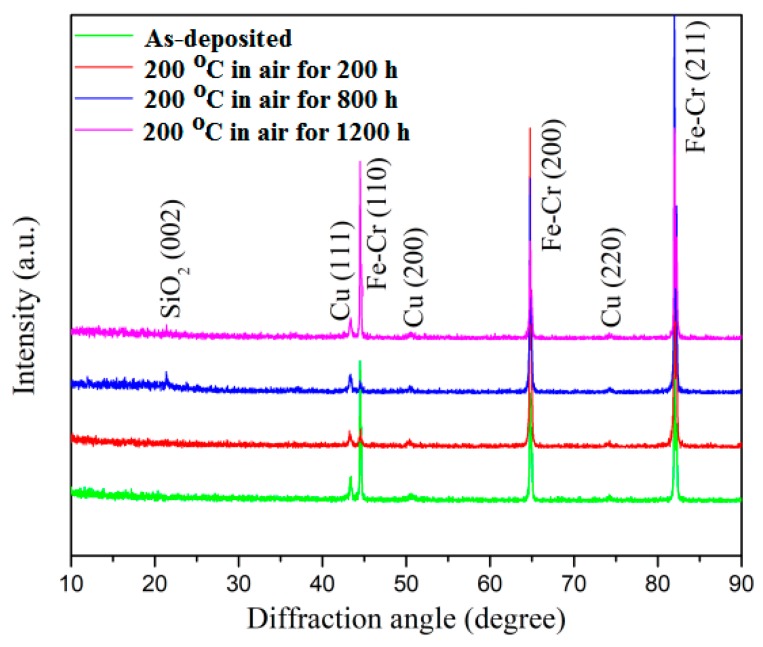
XRD spectra of the SSC as-deposited and after heat treatment.

Figure 7 shows the XRD patterns of the coating at 200, 800 and 1200 h after heat treatment. Compared with the as-deposited XRD pattern, all Cu diffraction peaks were retained, and no CuO diffraction peaks were observed. The SiO_2_ peak was found in the XRD pattern, indicating that certain oxides such as SiO_2_, appeared in AlSiN at high temperatures. Emittance is very sensitive to the oxidation of the coating. Oxidation on the surface of AlSiN causes changes in emittance. But Al_2_O_3_ or SiO_2_ has a lower refractive index and is the material of choice for anti-reflection layers. Therefore, the oxide generated by AlSiN plays a self-healing role on the coating. At the same time, SiO_2_ and Al_2_O_3_ have high thermal stability [27]. The AlSiN effectively prevents the penetration of oxygen to prevent oxidation of the LMVF and Cu. This characteristic was also found by Rebouta et al. preparing AlSiN/AlSiON coatings heated for 2500 h [25].

## 4. Conclusions

We use magnetron sputtering to prepare an oxygen-free coating of Cu/Ti_x_Si_y_N/AlSiN on a stainless steel substrate. This coating uses a single target to prepare each layer, which simplifies the process, has good process repeatability and a promising future market. Cu/Ti_x_Si_y_N/AlSiN has good optical properties (average absorption of 0.941 and emittance of 0.058) and excellent thermal stability. The PC of SSC is 0.0365 when heated in air at 200 °C for 1200 h. The Cu buffer layer plays an important role in reducing the emittance loss of the SSC deposited on the stainless steel substrate. AlSiN undergoes imperceptible oxidation in the heat treatment test and plays a self-healing function on the absorption of the coating, therefore it can effectively prevent the coating from oxidizing.

## Figures and Tables

**Table 1:** Optimized sputtering process parameters for the Cu/Ti_x_Si_y_N/AlSiN tandem absorber.

Layer	Flow Rate (sccm)		Sputtering Pressure (Pa)	Deposition Voltage (V)	Average Current (A)	Deposition Time (min)	Thickness (nm)
	Ar	N_2_					
Cu	40	0	0.6	368	0.5	15	100
Ti_x_Si_y_N_H_	40	25	1.0	470	0.6	10	80
Ti_x_Si_y_N_L_	40	40	1.0	460	0.5	10	70
AlSiN	40	40	0.6	510	0.5	7	70

**Table 2:** The absorptance, emittance, and solar selectivity values.

Layer	Absorptance (α)	Emittance (ε)	Solar Selectivity (S)
Cu	0.317	0.063	5.03
Cu/Ti_x_Si_y_N_H_	0.786	0.069	11.39
Cu/Ti_x_Si_y_N_H_-Ti_x_Si_y_N_L_	0.827	0.074	11.18
Cu/Ti_x_Si_y_N_H_-Ti_x_Si_y_N_L_/AlSiN	0.934	0.044	21.23
Ti_x_Si_y_N_H_-Ti_x_Si_y_N_L_/AlSiN	0.958	0.150	6.39

**Table 3:** The optical characteristics of SSC, which were deposited under the same process.

Sample #	Absorptance (α)	Emittance (ε)	Solar Selectivity (α/ε)
1	0.948	0.053	17.89
2	0.945	0.054	17.50
3	0.947	0.075	12.63
4	0.942	0.068	13.85
5	0.943	0.047	20.06
6	0.932	0.053	17.58
7	0.940	0.070	13.43
8	0.934	0.044	21.23
Average	0.941	0.058	16.23
Standard deviation	0.0058	0.0107	2.9581

**Table 4:** The optical characteristics of coating after heat treatment.

Heat Treatment in Air at 200 °C (h)	Absorptance (α)	Emittance (ε)	PC
As-deposited	0.948	0.053	-
200	0.943	0.056	0.0065
400	0.939	0.058	0.0115
800	0.932	0.065	0.022
1200	0.923	0.076	0.0365
